# An improved method for rapid generation of unmarked *Pseudomonas aeruginosa *deletion mutants

**DOI:** 10.1186/1471-2180-5-30

**Published:** 2005-05-23

**Authors:** Kyoung-Hee Choi, Herbert P Schweizer

**Affiliations:** 1 Department of Microbiology, Immunology and Pathology, Colorado State University, Fort Collins, Colorado, USA

## Abstract

**Background:**

Traditional gene replacement procedures are still time-consuming. They usually necessitate cloning of the gene to be mutated, insertional inactivation of the gene with an antibiotic resistance cassette and exchange of the plasmid-borne mutant allele with the bacterial chromosome. PCR and recombinational technologies can be exploited to substantially accelerate virtually all steps involved in the gene replacement process.

**Results:**

We describe a method for rapid generation of unmarked *P. aeruginosa *deletion mutants. Three partially overlapping DNA fragments are amplified and then spliced together *in vitro *by overlap extension PCR. The resulting DNA fragment is cloned *in vitro *into the Gateway vector pDONR221 and then recombined into the Gateway-compatible gene replacement vector pEX18ApGW. The plasmid-borne deletions are next transferred to the *P. aeruginosa *chromosome by homologous recombination. Unmarked deletion mutants are finally obtained by Flp-mediated excision of the antibiotic resistance marker. The method was applied to deletion of 25 *P. aeruginosa *genes encoding transcriptional regulators of the GntR family.

**Conclusion:**

While maintaining the key features of traditional gene replacement procedures, for example, suicide delivery vectors, antibiotic resistance selection and sucrose counterselection, the method described here is considerably faster due to streamlining of some of the key steps involved in the process, especially plasmid-borne mutant allele construction and its transfer into the target host. With appropriate modifications, the method should be applicable to other bacteria.

## Background

The elucidation of the *P. aeruginosa *strain PAO1 genome sequence in 2000 [1] opened the doors for genome- and proteome-scale research with this important opportunistic pathogen. These include the availability of commercial (Affymetrix, Santa Clara, CA) *P. aeruginosa *GeneChips^® ^for transcriptome analyses [2], as well as the *P. aeruginosa *PAO1 gene collection for high-throughput expression and purification of recombinant proteins [3]. A comprehensive transposon mutant library was generated containing over 30,000 sequence-defined mutants, corresponding to an average of five insertions per gene [4].

However, despite the development of many genetic tools for *P. aeruginosa *over the past decade [5,6], isolation of defined deletion mutants is still a relatively tedious process, which relies on construction of deletion alleles, most often tagged with an antibiotic resistance gene, on a suicide plasmid, followed by recombination of the plasmid-borne deletions into the chromosome, usually after conjugal transfer of the suicide plasmid (reviewed in [6]). Merodiploids arising from chromosomal integration of the suicide plasmid are resolved by utilization of a counterselectable marker, most often *Bacillus subtilis sacB *(sucrose counterselection) [7] or, less frequently, *rpsL *(streptomycin counterselection)[8]. In cases where the antibiotic selection markers are flanked by site specific recombination sites, e.g., the Flp recombinase target (*FRT*) [9] or Cre recombinase (*loxP*) [10] sites, they can subsequently be deleted from the chromosome, resulting in unmarked deletion mutants.

Unfortunately, the previously described method for one-step inactivation of chromosomal genes in *Escherichia coli *using PCR products [11] does not work in *P. aeruginosa*, although we have used a modification [12] of this approach where the target gene is first cloned into a plasmid, followed by λ RED-mediated recombination of a PCR-generated mutated copy of the gene [13]. Here, we describe a more streamlined method for generation of chromosomal deletion mutants. In this method, a "mutant" fragment containing the 5' and 3' flanking regions of the target gene plus the antibiotic resistance gene is assembled by a modified [14] PCR overlap extension protocol [15] and then cloned into a suicide vector using the Gateway recombinational cloning system, similarly to a previously described method [16]. The plasmid-borne deletion allele is then transferred to *P. aeruginosa *utilizing a rapid transformation procedure and recombined into the chromosome. The transformants are often merodiploids which are then resolved by sucrose counterselection against the undesired wild-type sequences. The antibiotic resistance marker is subsequently deleted from the chromosome by Flp recombinase.

## Results

### Construction of a Gateway-compatible suicide vector

**Figure 1:**
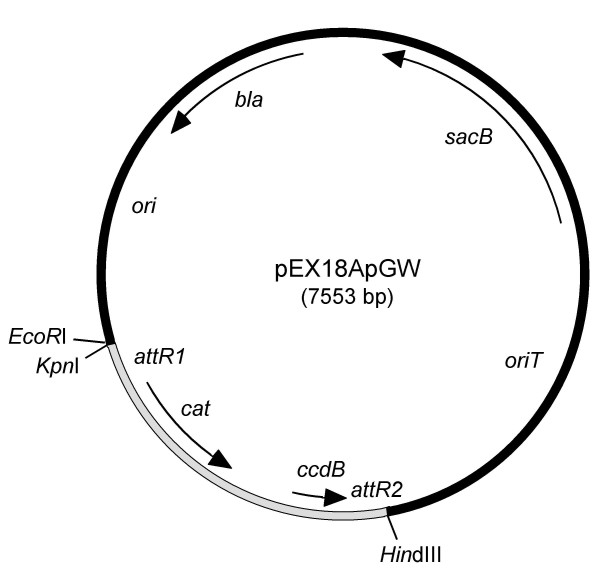
**Map of the Gateway-compatible pEX18ApGW**. This vector was derived by cloning a Gateway conversion fragment (grey) into the multiple cloning site of pEX18Ap (black). Only selected restriction enzyme cleavage sites are shown. Abbreviations: *attR1 *and *attR2*, bacteriophage λ recombination sites; *bla*, β-lactamase-encoding gene; *cat*, chloramphenicol acetyl transferase-encoding gene; *ccdB*, gene encoding gyrase-modifying enzyme (CcdB poisons host DNA gyrase by forming a covalent complex with the DNA gyrase A subunit and thus serves as a counter-selectable marker in *gyrA*^+ ^cloning hosts [25]); *ori*, ColE1-derived replication origin; *oriT*, origin of conjugal transfer; *sacB*, *Bacillus subtilis *levansucrase-encoding gene. The sequence of this plasmid was deposited in GenBank and assigned accession number AY928469.

We previously described the pEX family of relatively small, fully sequenced suicide plasmids which are now widely used for gene replacement experiments in *P. aeruginosa *and related bacteria [9]. Wolfgang et al. [16] recently described a Gateway-compatible pEX18Gm derivative. Here, we modified pEX18Ap for use as a Gateway destination vector for cloning of mutations marked with a gentamycin resistance (Gm^r^) encoding gene. We prefer using the Gm^r ^marker because it is more easily transferable between *P. aeruginosa *chromosomes by transformation with chromosomal DNA fragments when compared to other selection markers [17]. The resulting vector pEX18ApGW (Fig. 1) contains the functional sequences for recombination-based cloning (*attR1 *and *attR2*) and the *ccdB *counterselection marker, while maintaining the *sacB *counterselection marker for downstream resolution of merodiploids.

### Generation of a mutant fragment by PCR overlap extension

The amplification of each mutant fragment was achieved in a two step PCR that required 8 PCR primers, 4 of which were unique for each fragment to be mutated and 4 of which were common primers (Table 1). The gene-specific portions of the unique primers were designed to yield T_m_'s of 55–57°C and PCR fragments ranging from ~200 to 600 bp. These parameters enabled utilization of the same PCR conditions for different target genes, while at the same time provided for amplification of sufficient chromosomal DNA to allow for efficient homologous recombination of plasmid-borne sequence with the chromosome.

**Figure 2:**
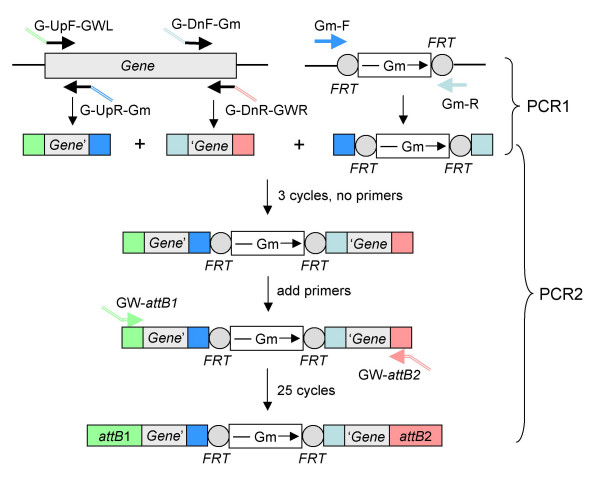
**Schematic illustration of mutant fragment generation by overlap extension PCR**. During first round PCR (PCR1), the 5' and 3' ends of the target genes, as well as the gentamycin (Gm) resistance cassette are amplified using four gene-specific primers (G-UpF-GWL, G-UpR-Gm, G-DnF-Gm and G-DnR-GWR) and the common Gm-specific primers (Gm-F and Gm-R). This generates three fragments with partial overlaps either to each other (indicated by the blue boxes signifying Gm overlap) or the *attB1 *and *attB2 *λ recombination sites (indicated by the green and pink boxes). These purified fragments are then assembled *in vitro *by overlap extension during second round PCR (PCR2) using common primers GW-*attB*1 and GW-*attB*2, resulting in a recombination-proficient mutant PCR fragment.

**Figure 3:**
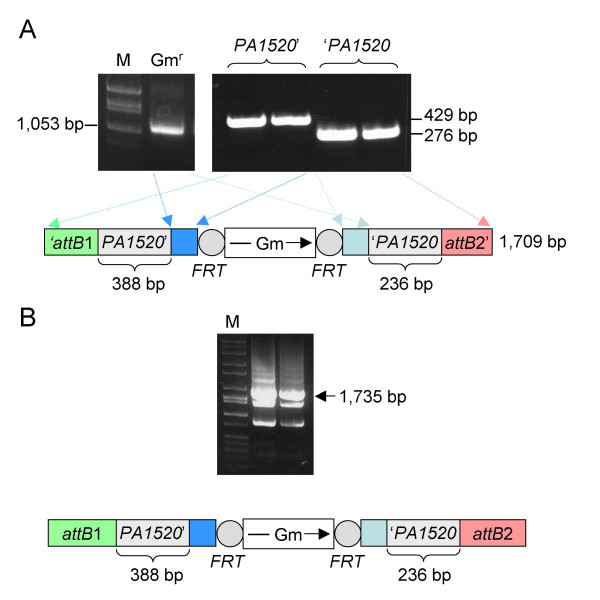
**PCR amplification of the Gm *FRT *cassette, *PA1520 *gene fragments and the overlap extension product**. **A. **First round PCR fragments. The left panel illustrates amplification of the 1,053-bp Gm^r ^fragment from pPS856 which contains 24 bp (right) and 25 bp (left) overlaps with the *PA1520*' and '*PA1520 *fragments (blue boxes). The right panel illustrates amplification of the *PA1520*' and '*PA1520 *fragments. The 5' fragment contains 388 bp of chromosomal DNA, 25 bp overlapping the left side of the Gm^r ^fragment and 16 bp overlapping the GW-*attB1 *primer. Similarly, the 3' fragment contains 236 bp of chromosomal DNA, 24 bp overlapping the right side of the Gm^r ^fragment and 16 bp overlapping the GW-*attB2 *primer. The sequences of the gene-specific and common primers are listed in Table 1. **B. **Second round PCR. The purified fragments shown in panel **A **were used in the second round PCR illustrated in Fig. 2 to derive the indicated *attB1*-*PA1520*'-*FRT*-Gm-*FRT*-'*PA1520*-*attB2 *fragment. The entire 50 μl second round PCR reaction was subjected to agarose gel electrophoresis. The desired DNA fragment constituting the major product marked by the arrow was excised from the gel, purified and then used for the BP clonase reaction. Lanes labeled M in both panels contained Hi-Lo molecular size markers from Minnesota Molecular (Minneapolis, MN).

The basic strategy for generation of the mutant fragment by PCR overlap extension is illustrated in Fig. 2. In PCR1, the four gene-specific primers are used to amplify the 5' and 3' regions of the target gene. Simultaneously, two of the common primers, Gm-F and Gm-R, are used to amplify the Gm^r ^cassette flanked by *FRT *sites from a pPS856 template. Since the Gm^r ^fragment is used for all constructs, it can be prepared in larger amounts ahead of time and stored until needed. Fig. 3A illustrates typical results of PCR1 utilizing the example of *P. aeruginosa *gene *PA1520*, encoding a putative transcriptional regulator of the GntR family. When we adhered to the prescribed PCR conditions, the amplified fragments were very clean. It was especially prudent to follow the conditions for PCR amplification of the Gm^r ^*FRT *fragment since the *FRT *sites possess significant secondary structures.

The PCR fragments were gel-purified and equal amounts (50 ng) of each were combined for PCR2. PCR2 was allowed to proceed for 3 cycles in the absence of exogenous primers, which for *PA1520 *yields the intermittent 1,709 bp '*attB1*-*PA1520*'-*FRT*-Gm-*FRT*-'*PA1520*-*attB2*' fragment illustrated in Fig 3A. The common Gateway primers GW-*attB1 *and GW-*attB2 *were then added and the PCR continued for another 25 cycles to obtain the final, recombination-proficient DNA fragment, which constituted the predominant PCR product at this stage (Fig. 3B). For *PA1520*, the resulting 1,735 bp *attB1*-*PA1520*'-*FRT*-Gm-*FRT*-'*PA1520*-*attB2 *fragment was gel-purified and used for the BP clonase reaction for construction of an entry clone.

### BP and LR clonase reactions

**Figure 4:**
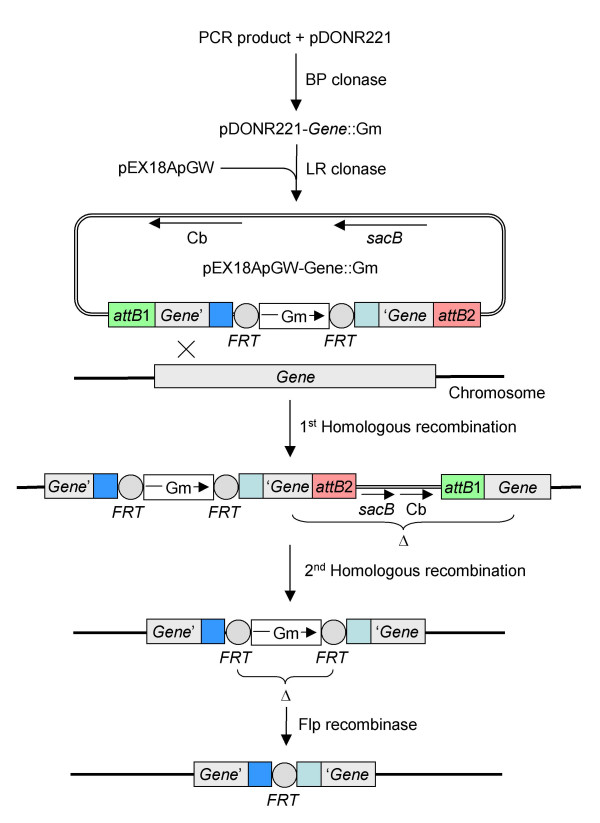
**Gateway-recombinational cloning and return of the plasmid-borne deletion allele to the *P. aeruginosa *chromosome**. The mutant DNA fragment generated by overlap extension PCR is first cloned into pDONR221 via the BP clonase reaction to create the entry clone pDONR221-*Gene*::Gm, which then serves as the substrate for LR clonase-mediated recombination into the destination vector pEX18ApGW. The resulting suicide vector pEX18ApGW-*Gene*::Gm is then transferred to *P. aeruginosa *and the plasmid-borne deletion mutation is exchanged with the chromosome to generate the desired deletion mutant. Please note that, as discussed in the text, gene replacement by double-crossover can occur quite frequently, but it can also be a rare event in which case allele exchange happens in two steps involving homologous recombination. First, the suicide plasmid is integrated via a single-crossover event resulting in generation of a merodiploid containing the wild-type and mutant allele. Second, the merodiploid state is resolved by *sacB*-mediated sucrose counterselection in the presence of gentamycin, resulting in generation of the illustrated chromosomal deletion mutant. An unmarked mutant is then obtained after Flp recombinase-mediated excision of the Gm marker.

The BP and LR clonase reactions (Fig. 4) were performed according to Invitrogen's (Carlsbad, CA) Gateway cloning manual. For the BP clonase reaction pDONR221 was used, but only half of the recommended BP clonase enzyme mix. Gm^r ^and kanamycin resistant (Km^r^) DH5α or HPS1 transformants were selected and the presence of the correct plasmids was confirmed by *Xba*I digestion (the *FRT *sites flanking the Gm^r ^gene each contain a single *Xba*I site). The insert of a correct pDONR-*Gene*::Gm entry clone was then recombined into the destination vector pEX18ApGW using the LR clonase protocol, again using only half of the recommended amount of LR clonase mix. Recombinants were transformed into DH5α and Ap^r ^colonies were selected. We noticed that, at least under the conditions we used, many transformants exhibited a Ap^r ^Km^r ^phenotype, indicating that they either contained both pDONR-*Gene*::Gm and pEX18ApGW-*Gene*::Gm or, more likely, a cointegrate of both plasmids. The presence of the correct pEX18ApGW-*Gene*::Gm in transformants that were only Ap^r ^was confirmed by *Xba*I digestion.

### Gene replacement

**Figure 5:**
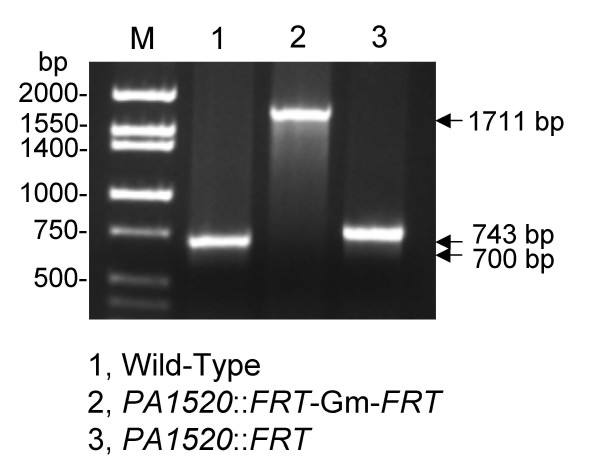
**PCR analysis of marked and unmarked *P. aeruginosa PA1520 *deletion strains**. Colony PCR was performed on either wild-type PAO1 and its *PA1520 *mutant derivatives, either containing a marked (*PA1520*::*FRT*-Gm-*FRT*) or unmarked (*PA1520*::*FRT*) *PA1520 *deletion. The sizes of the expected PCR fragments are indicated. Note that the short deletion removes 41 bp of the *PA1520 *coding sequence, corresponding to codons 131 to 145, but replaces these sequences with a 85 bp *FRT *scar. Lane M contained Hi-Lo molecular size markers of the indicated sizes from Minnesota Molecular.

To expedite gene replacement, an improved electroporation method [17] was used for transfer of the suicide plasmid pEX18ApGW-Gene::Gm into *P. aeruginosa *instead of the traditionally employed conjugation method. Although after transfer into *P. aeruginosa *double cross-over events can occur frequently (>50%), they can also be rare (<1–5%). In the latter instance, merodiploids formed via integration of the suicide plasmid by a single cross-over event (Fig. 4). The merodiploid state was then resolved via sucrose selection in the presence of gentamycin, resulting in deletion of the wild type gene. For generation of unmarked deletion mutants, the Gm^r ^marker was subsequently removed by deletion of a 967 bp fragment using Flp recombinase. The presence of the desired deletion in either the marked or unmarked *PA1520 *mutant was verified by colony PCR utilizing the gene-specific up and down primers (Fig. 5).

### Deletion of 25 genes encoding transcriptional regulators of the GntR family

**Figure 6:**
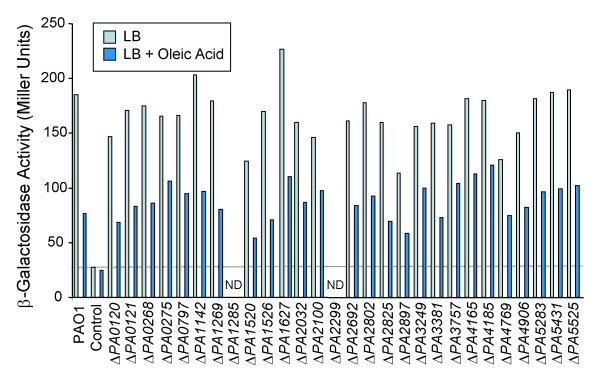
**Effect of deletions of *P. aeruginosa *GntR homologs on *fabA *expression**. The indicated genes were deleted in strain PAO1 containing a chromosomally integrated *fabA'-lacZ *fusion (labelled PAO1). The control was PAO1 with a *fabA*Δ30'-*lacZ *fusion; this strain harbours the same *fabA'-lacZ *fusion but contains a deletion of the putative 30 nucleotide activator-binding site in the *fabA *promoter region. Strains were grown to mid-log phase in LB medium containing 0.05% Brij 58 +/- 0.05% oleic acid and β-galactosidase expression was monitored in triplicate samples. The dotted line marks expression levels observed in the putative activator binding-site mutant and similar levels would be expected in an activator mutant.

We are interested in characterizing the activator involved in regulation of expression of the *fabAB *operon, which encodes two essential enzymes of *de novo *fatty acid biosynthesis [18]. Expression of the *fabAB *operon is repressed by exogenously added fatty acids, most effectively by oleic acid, and preliminary results from our laboratory indicate that this may be due to relief of activation by a transcriptional regulator which binds to a 30 nucleotide site located in the *fabAB *promoter region (Choi and Schweizer, unpublished results). This 30 bp region is only found and conserved in *Pseudomonas *spp. Its deletion dramatically reduces transcription of the *fabAB *operon and the residual transcriptional activity is no longer fatty acid responsive (Fig. 6). In *E. coli*, transcriptional activation of the unlinked *fabA *and *fabB *genes is achieved by FadR, a regulator belonging to a group of transcriptional regulators with the GntR family signature [19,20]. The PAO1 chromosome encodes more than 30 regulatory proteins with this signature. Of these, 27 have not been assigned function. Using the methodology described above for one of these genes, *PA1520*, we deleted 25 of the 27 genes (we could not delete the presumably essential *PA1285 *and *PA2299*) in less than 4 weeks in strain PAO1 with a chromosomally integrated *fabA'-lacZ *transcriptional fusion and analyzed β-galactosidase expression in the resulting mutant strains (Fig. 6). In exponential phase cells, the overall pattern of *fabA *expression was indistinguishable from the parental strain in all 25 mutants, indicating that none of these genes encodes the *fabAB *activator. We would have expected an expression pattern similar to that observed in the PAO1::*fabA*Δ30'-*lacZ *control strain which contains a deletion of the 30 nucleotide putative activator-binding site.

## Discussion

In our experience, the method described here for generation of unmarked deletion mutants is the fastest available to date. From start to finish, an individual unmarked *P. aeruginosa *mutant can be generated and characterized in ~14 days, which is up to one week faster than using our previously described method [9]. However, the main advantage of the method does not lie in individual mutant generation but in the speed by which collective deletion mutant pools can be generated. For example, we isolated unmarked deletions in 25 of the 27 genes encoding transcriptional regulators of the GntR family in less than 4 weeks. Two genes could not be deleted, presumably because they encode essential proteins. Besides speed, what are some of the other unique features of this method?

First, the deletion of gene sequences is not restricted by the natural availability or synthetic generation of restriction sites that are subsequently used to delete intervening sequences. With proper primer location, the PCR-based method allows clean deletion of more-or-less the entire coding sequence without fear of generating truncated genes which potentially produce products with undesired side effects.

Second, Flp excision of the antibiotic resistance gene cassette leaves behind a short 85 bp *FRT *scar which does not exhibit any known polar effects on downstream genes. The method is therefore uniquely suited to study genes organized in polycistronic units. Flp excision is a very straightforward procedure with efficiencies approaching 100% [9].

Third, the method is economical, especially in a more high-throughput format. PCR overlap extension requires only four gene specific primers of reasonable length which keeps oligonucleotide costs to a minimum. In comparison to traditional methods, it also requires fewer hands-on steps and gene-specific reagents (e.g., restriction enzymes).

## Conclusion

Although not yet as fast as the PCR fragment-directed gene replacement method described for *E. coli*, the PCR- and recombinational cloning based method described opens the door for high-throughput generation of *P. aeruginosa *deletion mutants on a genome-wide scale. With appropriate modifications, i.e., choice of appropriate selection markers and Gateway-compatible gene replacement vectors, the method should be applicable to the many other bacteria in which the pEX vector system can be applied [6].

## Methods

### Media

*Escherichia coli *and *P. aeruginosa *strains were maintained on Luria-Bertani medium (LB; 10 g per liter tryptone, 5 g per liter yeast extract, 10 g per liter NaCl; Becton, Dickinson & Co., Sparks, MD). For plasmid maintenance in *E. coli*, the medium was supplemented with 100 μg per ml ampicillin, 25 μg per ml chloramphenicol, 35 μg per ml kanamycin or 15 μg per ml gentamycin. For marker selection in *P. aeruginosa*, 200 μg per ml carbenicillin and 30 μg per ml of gentamycin was used, as appropriate.

### β-galactosidase activity assays

Cells were grown at 37°C with shaking to exponential phase (optical density at 540 nm ~0.4–0.8) in LB medium with 0.05% Brij 58 +/- 0.05% oleic acid. β-galactosidase activity was measured in chloroform/sodium dodecyl sulfate-permeabilized cells and its activity calculated as previously described [21].

### Standard DNA procedures

Routine procedures were employed for manipulation of DNA [22]. Plasmid DNAs were isolated using the QIAprep Mini-spin kit (Qiagen, Valencia, CA) and *P. aeruginosa *chromosomal DNA was obtained using the QIAamp DNA Mini Kit. DNA fragments were purified from agarose gels utilizing the QIAquick gel extraction kit.

### Construction of the Gateway-compatible gene replacement vector pEX18ApGW

The previously described gene replacement vector pEX18Ap [9] was modified by cloning of a 1,755 bp *Hin*dIII-*Kpn*I fragment from pUC18-mini-Tn*7*T-Gm-GW (GenBank accession number AY737004), followed by transformation into the *gyrA *strain DB3.1 (Invitrogen).

### 1^st ^round PCRs

#### PCR-amplification of the gentamycin resistance gene cassette

A 50 μl PCR reaction contained 5 ng pPS856 [9] template DNA, 1x HiFi Platinum *Taq *buffer, 2 mM MgSO_4_, 200 μM dNTPs, 0.2 μM of Gm-F and Gm-R (Table 1) and 5 units of HiFi Platinum *Taq *polymerase (Invitrogen). Cycle conditions were 95°C for 2 min, followed by 30 cycles of 94°C for 30 s, 50°C for 30 s, and 68°C for 1 min 30 s, and a final extension at 68°C for 7 min. The resulting 1,053 bp PCR product was purified by agarose gel electrophoresis and its concentration determined spectrophotometrically (A_260 nm_) in an Eppendorf Biophotometer (Hamburg, Germany) and using the 50–2000 μl Eppendorf UVettes

#### PCR-amplification of 5' and 3' gene fragments

Two 50 μl PCR reactions were prepared. The first reaction contained 20 ng chromosomal template DNA, 1x HiFi Platinum *Taq *buffer, 2 mM MgSO_4_, 5% DMSO, 200 μM dNTPs, 0.8 μM of PA1520-UpF-GWL and PA1520-UpR-Gm (Table 1) and 5 units of Platinum *Taq *polymerase. The second reaction contained the same components as the first, but 0.8 μM of PA1520-DnF-Gm and PA1520-DnR-GWR (Table 1). Cycle conditions were 94°C for 5 min, followed by 30 cycles of 94°C for 30 s, 56°C for 30 s, and 68°C for 30 s, and a final extension at 68°C for 10 min. The resulting PCR products were purified by agarose gel electrophoresis and their concentrations determined spectrophotometrically (A_260 nm_).

### 2^nd ^round PCR

A 50 μl PCR reaction contained 50 ng each of the *PA1520 *5' and 3' purified template DNAs, and 50 ng of *FRT*-Gm-*FRT *template DNA prepared during 1^st ^round PCR. The reaction mix also contained 1x HiFi Platinum *Taq *buffer, 2 mM MgSO_4_, 5% DMSO and 200 μM dNTPs, and 5 units of HiFi Platinum *Taq *polymerase. After an initial denaturation at 94°C for 2 min, 3 cycles of 94°C for 30 s, 55°C for 30 s, and 68°C for 1 min were run without added primers. The third cycle was paused at 30 s of the 68°C extension, primers GW-*attB1 *and GW-*attB2 *were added to 0.2 μM each, and the cycle was then finished by another 30 s extension at 68°C. The PCR was completed by 25 cycles of 94°C for 30 s, 56°C for 30 s, and 68°C for 5 min, and a final extension at 68°C for 10 min. The resulting major PCR product was purified by agarose gel electrophoresis and its concentrations determined spectrophotometrically (A_260 nm_). The identity of the PCR fragment was confirmed by *Xba*I digestion (each *FRT *site of the *FRT*-Gm*-FRT *fragment contains a *Xba*I site).

#### BP and LR clonase reactions

The BP and LR clonase reactions for recombinational transfer of the PCR product into pDONR221 and pEX18ApGW, respectively, were performed as described in Invitrogen's Gateway cloning manual, but using only half of the recommended amounts of BP and LR clonase mixes and either *E. coli *DH5α [23] or HPS1 [24] as host strains. The presence of the correct fragments in transformants obtained with DNA from either clonase reaction was verified by digestion with *Xba*I because each *FRT *site flanking the Gm^r ^gene contains a *Xba*I site. However, before plasmid isolation from transformants obtained with DNA from the LR clonase reaction, 25–50 transformants were i) patched on LB+Km and LB+Ap plates, and ii) simultaneously purified for single colonies on LB+Ap plates. This was necessary to distinguish between those colonies containing only the desired pEX18ApGW-*Gene*::Gm from those containing this plasmid and the frequently contaminating pDONR-*Gene*::Gm (pEX18Ap-derived plasmids confer Ap^r ^and pDONR plasmids confer Km^r^). Plasmids were then isolated from Ap^r ^Km^s ^transformants and analyzed by *Xba*I digestion. In those extremely rare instances where all patched transformants contained a mixed plasmid population (<1% of to date performed LR clonase reactions), retransformation with Ap^r ^selection was necessary to obtain colonies containing only pEX18ApGW-*Gene*::Gm.

#### Transfer of plasmid-borne deletions to the *P. aeruginosa *chromosome

A rapid electroporation method described elsewhere [17] was used to transfer the pEX18ApGW-borne deletion mutations to *P. aeruginosa*. Briefly, 6 ml of an overnight culture grown in LB medium was harvested in 4 microcentrifuge tubes by centrifugation (1–2 min, 16,000 × *g*) at room temperature. Each cell pellet was washed twice with 1 ml of room temperature 300 mM sucrose and they were then combined in a total of 100 μl 300 mM sucrose. For electroporation, 300-500 ng of plasmid DNA was mixed with 100 μl of electrocompetent cells and transferred to a 2 mm gap width electroporation cuvette. After applying a pulse (settings: 25 μF; 200 Ohm; 2.5 kV on a Bio-Rad GenePulserXcell™), 1 ml of LB medium was added at once, and the cells were transferred to a 17 × 100 mm glass or polystyrene tube and shaken for 1 h at 37°C. The cells were then harvested in a microcentrifuge tube. 800 μl of the supernatant was discarded and the cell pellet resuspended in the residual medium. The entire mixture was then plated on two LB plates containing 30 μg per ml Gm (LB+Gm30). The plates were incubated at 37°C until colonies appeared (usually within 24 h). Under these conditions, the transformation efficiencies were generally 30–100 transformants per μg of DNA. A few colonies were patched on LB+Gm30 plates and LB+Cb200 plates to differentiate single- from double cross-over events. To ascertain resolution of merodiploids, Gm^r ^colonies were struck for single colonies on LB+Gm30 plates containing 5% sucrose. Gm^r ^colonies from the LB-Gm-sucrose plates were patched onto LB+Gm30+5% sucrose, as well as LB plates with 200 μg per ml carbenicillin (LB+Cb200). Colonies growing on the LB-Gm-sucrose, but not on the LB-carbenicillin plates were considered putative deletion mutants. The presence of the correct mutations was verified by colony PCR. To do this, a single, large colony (or the equivalent from a cell patch) was picked from a LB-Gm-sucrose plate, transferred to 30 μl H_2_O in a microcentrifuge tube and boiled for 5 min. Cell debris was removed by centrifugation in a microcentrifuge fuge (2 min; 16,000 × *g*), and the supernatant was transferred to a fresh tube which was placed on ice. 5 μl of the supernatant was used as source of template DNA in a 50 μl PCR reaction containing *Taq *buffer, 1.5 mM MgSO_4_, 5% DMSO, 0.6 μM each of PA1520-UpF-GWL and PA1520-DnR-GWR, 200 μM dNTPs and 5 units *Taq *polymerase (Fermentas, Hanover, MD). Cycle conditions were 95°C for 5 min, followed by 30 cycles of 95°C for 45 s, 55°C for 30 s, and 72°C for 2 min, and a final extension at 72°C for 10 min. PCR products were analyzed by agarose gel electrophoresis.

#### Flp-mediated marker excision

Electrocompetent cells of the newly constructed mutant strain were prepared as described in the preceding paragraph and transformed with 20 ng of pFLP2 [9] DNA as described above. After phenotypic expression at 37°C for 1 h, the cell suspension was diluted 1:1,000 and 1:10,000 with either LB or 0.9% NaCl, and 50 μl aliquots were plated on LB+Cb200 plates and incubated at 37°C until colonies appeared. Transformants were purified for single colonies on LB+Cb200 plates. Ten single colonies were tested for antibiotic-susceptibility on LB ± Gm30 plates and on a LB+Cb200 plate. Two Gm^s ^Cb^r ^isolates were struck for single colonies onto a LB+5% sucrose plate and incubated at 37°C until sucrose-resistant colonies appeared. Ten sucrose-resistant colonies were retested on a LB+5% sucrose (master) plate and a LB+Cb200 plate. Finally, two sucrose-resistant and Cb^s ^colonies were struck on LB plates without antibiotics, and their Cb^s ^and Gm^s ^phenotypes confirmed by patching on LB ± Cb200 and LB ± Gm30 plates. Deletion of the Gm^r ^marker was assessed by colony PCR utilizing the conditions and primers described in the preceding paragraph

## Authors' contributions

KHC, performance of experiments, writing of manuscript.

HPS, design and coordination of the study, data evaluation, direct supervision of experimental work, writing of manuscript.

All authors read and approved the final manuscript.

## Supplementary Material

Additional File 1 Table 2 – Sequences of PCR primers used to amplify genes encoding transcriptional regulators of the GntR family Click here for file

## Figures and Tables

**Table 1:** PCR oligonucleotides

**Name**	**Sequence (5'→3')^1^**
Gene-specific primers	

G-UpF-GWL^2^	TACAAAAAAGCAGGCTccgatcagttgcaacacatc
G-UpR-Gm^2^	TCAGAGCGCTTTTGAAGCTAATTCGttgttcctgcaccaccatct
G-DnF-Gm^2^	AGGAACTTCAAGATCCCCAATTCGccggcgagttccacctgaa
G-DnR-GWR^2^	TACAAGAAAGCTGGGTggttgagcttgctgtcgata

Common primers	

Gm-F	CGAATTAGCTTCAAAAGCGCTCTGA
Gm-R	CGAATTGGGGATCTTGAAGTTCCT
GW-*attB*1	GGGGACAAGTTTGTACAAAAAAGCAGGCT
GW-*attB*2	GGGGACCACTTTGTACAAGAAAGCTGGGT

^1^Sequences in capital letters are common for all genes amplified and overlap with the Gm or *attB *primer sequences. Lower-case letters indicate gene-specific sequences, here *PA1520*. PCR oligonucleotide sequences for all 27 genes amplified in this study are given in Table 2 (see Additional file 1).

^2^G, gene specific sequence, here *PA1520*.
