# Supplementary material for: An improved method for rapid generation of unmarked Pseudomonas aeruginosa deletion mutants
Source: BMC Microbiol. 2005 May 23;5:30. doi: 10.1186/1471-2180-5-30 (PMC1173109; doi:10.1186/1471-2180-5-30)
Supplement: Additional File 1 — Table 2 – Sequences of PCR primers used to amplify genes encoding transcriptional regulators of the GntR family [file 1471-2180-5-30-S1.doc]

**Table 2 - Sequences of PCR primers used to amplify genes encoding transcriptional regulators of the GntR family**

| **Name** | **Sequence (5’ 🡪 3’)^1^** |
| --- | --- |
| PA0120-UpF-GWL  PA0120-UpR-Gm  PA0120-DnF-Gm  PA0120-DnR-GWR | TACAAAAAAGCAGGCTtcttcgtccaagctggtcaa  TCAGAGCGCTTTTGAAGCTAATTCGatgaacggctccagcgcata  AGGAACTTCAAGATCCCCAATTCGatcagcgaactgcggctgtt  TACAAGAAAGCTGGGTttgaggatgtgctggcgcat |
| PA0121-UpF-GWL  PA0121-UpR-Gm  PA0121-DnF-Gm  PA0121-DnR-GWR | TACAAAAAAGCAGGCTattcgaagcacgtacccagc  TCAGAGCGCTTTTGAAGCTAATTCGcacttcgctttccaccagca  AGGAACTTCAAGATCCCCAATTCGaactgaccgagaatcccttg  TACAAGAAAGCTGGGTccttcatgccgaattcctcg |
| PA0268-UpF-GWL  PA0268-UpR-Gm  PA0268-DnF-Gm  PA0268-DnR-GWR | TACAAAAAAGCAGGCTaactgcaactgatcctcgac  TCAGAGCGCTTTTGAAGCTAATTCGgtagaccagcttggcgttgt  AGGAACTTCAAGATCCCCAATTCGgatcatcgaggacgactacg  TACAAGAAAGCTGGGTtcgatgtcgtcccgttcgat |
| PA0275-UpF-GWL  PA0275-UpR-Gm  PA0275-DnF-Gm  PA0275-DnR-GWR | TACAAAAAAGCAGGCTtgaacgagcgtaccgactac  TCAGAGCGCTTTTGAAGCTAATTCGtacctggagtagctgggtgt  AGGAACTTCAAGATCCCCAATTCGctgctgatgagccacaagct  TACAAGAAAGCTGGGTaggtcgagaatctcgatgcc |
| PA0797-UpF-GWL  PA0797-UpR-Gm  PA0797-DnF-Gm  PA0797-DnR-GWR | TACAAAAAAGCAGGCTattcgaagcacgtacccagc  TCAGAGCGCTTTTGAAGCTAATTCGcacttcgctttccaccagca  AGGAACTTCAAGATCCCCAATTCGagaatcccttgctgcagggt  TACAAGAAAGCTGGGTgccttcatgacgaatgcctc |
| PA1142-UpF-GWL  PA1142-UpR-Gm  PA1142-DnF-Gm  PA1142-DnR-GWR | TACAAAAAAGCAGGCTtgacggtgatctaccagtcc  TCAGAGCGCTTTTGAAGCTAATTCGaccatcgagtggaagtggct  AGGAACTTCAAGATCCCCAATTCGgtgctgtacgtcgagcacta  TACAAGAAAGCTGGGTacctcgacgttgacctgtag |
| PA1269-UpF-GWL  PA1269-UpR-Gm  PA1269-DnF-Gm  PA1269-DnR-GWR | TACAAAAAAGCAGGCTatgctcaacgcccctaacct  TCAGAGCGCTTTTGAAGCTAATTCGttcggaaatacgcgtcacca  AGGAACTTCAAGATCCCCAATTCGagatgttcgaagtgcgggtg  TACAAGAAAGCTGGGTcgacgatatgtgcctcgatc |
| PA1285-UpF-GWL  PA1285-UpR-Gm  PA1285-DnF-Gm  PA1285-DnR-GWR | TACAAAAAAGCAGGCTcgacgagaggtacgggtata  TCAGAGCGCTTTTGAAGCTAATTCGtcggcgagttcggaaatgct  AGGAACTTCAAGATCCCCAATTCGgcaatctgcgagtgctggaa  TACAAGAAAGCTGGGTtcagtccagtcgctccagtt |
| PA1520-UpF-GWL  PA1520-UpR-Gm  PA1520-DnF-Gm  PA1520-DnR-GWR | Tacaaaaaagcaggctccgatcagttgcaacacatc  tcagagcgcttttgaagctaattcgttgttcctgcaccaccatct  aggaacttcaagatccccaattcgccggcgagttccacctgaa  tacaagaaagctgggtggttgagcttgctgtcgata |
| PA1526-UpF-GWL  PA1526-UpR-Gm  PA1526-DnF-Gm  PA1526-DnR-GWR | TACAAAAAAGCAGGCTcaaggaacgggaacgtatcc  TCAGAGCGCTTTTGAAGCTAATTCGgcgtagaggctttccacatg  AGGAACTTCAAGATCCCCAATTCGcgttcagctctacatcctgc  TACAAGAAAGCTGGGTactgccgaggaactggttca |
| PA2032-UpF-GWL  PA2032-UpR-Gm  PA2032-DnF-Gm  PA2032-DnR-GWR | TACAAAAAAGCAGGCTaacaggcatacaggctgctg  TCAGAGCGCTTTTGAAGCTAATTCGgcctttcagcgattgcatgg  AGGAACTTCAAGATCCCCAATTCGtggaacagtggccgatcaag  TACAAGAAAGCTGGGTcgcaggcagttccgatactt |
| PA2100-UpF-GWL  PA2100-UpR-Gm  PA2100-DnF-Gm  PA2100-DnR-GWR | TACAAAAAAGCAGGCTctgaagtacaaacggctggc  TCAGAGCGCTTTTGAAGCTAATTCGgcacagaatctgctggtgct  AGGAACTTCAAGATCCCCAATTCGtggatgaagaggggctactg  TACAAGAAAGCTGGGTgtactaaaggcctctgcggt |

| PA2299-UpF-GWL  PA2299-UpR-Gm  PA2299-DnF-Gm  PA2299-DnR-GWR | TACAAAAAAGCAGGCTtgtacagccagctcaaggag  TCAGAGCGCTTTTGAAGCTAATTCGtagcccatctgcgtcatgga  AGGAACTTCAAGATCCCCAATTCGttcgctggaagtcacctacc  TACAAGAAAGCTGGGTgaggtattcgaagtccagcg |
| --- | --- |
| PA2692-UpF-GWL  PA2692-UpR-Gm  PA2692-DnF-Gm  PA2692-DnR-GWR | TACAAAAAAGCAGGCTtcgaatacggcatccactgc  TCAGAGCGCTTTTGAAGCTAATTCGgagcacgctgatttcgttgg  AGGAACTTCAAGATCCCCAATTCGtcgacggcgacaaatcgatc  TACAAGAAAGCTGGGTcatccagcgcagcacttctt |
| PA2802-UpF-GWL  PA2802-UpR-Gm  PA2802-DnF-Gm  PA2802-DnR-GWR | TACAAAAAAGCAGGCTcaatctcgtcagtacctgcg  TCAGAGCGCTTTTGAAGCTAATTCGggcgctgacgtattccatga  AGGAACTTCAAGATCCCCAATTCGtgctgatcgaactgatcgcc  TACAAGAAAGCTGGGTccagaactcctggtcgaact |
| PA2825-UpF-GWL  PA2825-UpR-Gm  PA2825-DnF-Gm  PA2825-DnR-GWR | TACAAAAAAGCAGGCTgatggcgtcgaagaatcgct  TCAGAGCGCTTTTGAAGCTAATTCGagcaccagcatgaccaggta  AGGAACTTCAAGATCCCCAATTCGtcaagcgtctcgaacagctc  TACAAGAAAGCTGGGTctggtgcaggtcgaacatct |
| PA2897-UpF-GWL  PA2897-UpR-Gm  PA2897-DnF-Gm  PA2897-DnR-GWR | TACAAAAAAGCAGGCTgctgctttaccaacgcatcg  TCAGAGCGCTTTTGAAGCTAATTCGatgtggatcgcatccacgca  AGGAACTTCAAGATCCCCAATTCGcgaactgatgttcgaccagg  TACAAGAAAGCTGGGTgaactgctcggtgttgctga |
| PA3249-UpF-GWL  PA3249-UpR-Gm  PA3249-DnF-Gm  PA3249-DnR-GWR | TACAAAAAAGCAGGCTtcagcgagcagatcgtccat  TCAGAGCGCTTTTGAAGCTAATTCGctgaccatcgcatggaagtg  AGGAACTTCAAGATCCCCAATTCGtgtccagcgtctaccagatc  TACAAGAAAGCTGGGTaactccaggtcgcagtcgat |
| PA3381-UpF-GWL  PA3381-UpR-Gm  PA3381-DnF-Gm  PA3381-DnR-GWR | TACAAAAAAGCAGGCTgcacttgtctagacaaagcg  TCAGAGCGCTTTTGAAGCTAATTCGttcgagcaccagttcgtcga  AGGAACTTCAAGATCCCCAATTCGaactgaccaccctgcgttac  TACAAGAAAGCTGGGTgacctggtactggaagcgat |
| PA3757-UpF-GWL  PA3757-UpR-Gm  PA3757-DnF-Gm  PA3757-DnR-GWR | TACAAAAAAGCAGGCTatgaagacagcccacgacct  TCAGAGCGCTTTTGAAGCTAATTCGagacgcggcgtaatgaaggt  AGGAACTTCAAGATCCCCAATTCGctctccagcttcagcgagat  TACAAGAAAGCTGGGTatccgggtcatcagcagcat |
| PA4165-UpF-GWL  PA4165-UpR-Gm  PA4165-DnF-Gm  PA4165-DnR-GWR | TACAAAAAAGCAGGCTtcactgctcgagtcggtcaa  TCAGAGCGCTTTTGAAGCTAATTCGacgctccaggttgacgtagt  AGGAACTTCAAGATCCCCAATTCGgcatggatcatcgaggacga  TACAAGAAAGCTGGGTtcgccgtagagattgcgcat |
| PA4185-UpF-GWL  PA4185-UpR-Gm  PA4185-DnF-Gm  PA4185-DnR-GWR | TACAAAAAAGCAGGCTgatcctgagcatggaactgg  TCAGAGCGCTTTTGAAGCTAATTCGcgtggtggcgatgtagatca  AGGAACTTCAAGATCCCCAATTCGccaggaagatttccgcaagg  TACAAGAAAGCTGGGTatggcatcgacgatctggtc |
| PA4769-UpF-GWL  PA4769-UpR-Gm  PA4769-DnF-Gm  PA4769-DnR-GWR | TACAAAAAAGCAGGCTctgtcggatgacatcgttgc  TCAGAGCGCTTTTGAAGCTAATTCGtagtaggcacaggacccttc  AGGAACTTCAAGATCCCCAATTCGacaccatcaagggcctgttc  TACAAGAAAGCTGGGTcaggacctcctgcacatagt |
| PA4906-UpF-GWL  PA4906-UpR-Gm  PA4906-DnF-Gm  PA4906-DnR-GWR | TACAAAAAAGCAGGCTagctgcacaagctgatcgct  TCAGAGCGCTTTTGAAGCTAATTCGgccgttgtcgaacaatcggt  AGGAACTTCAAGATCCCCAATTCGtgaacatgcgtttccaccgc  TACAAGAAAGCTGGGTatcgaagtagtcggcgtagc |
| PA5283-UpF-GWL  PA5283-UpR-Gm  PA5283-DnF-Gm  PA5283-DnR-GWR | TACAAAAAAGCAGGCTgattccgtcgatacgccagt  TCAGAGCGCTTTTGAAGCTAATTCGcatggatatcgatcagccgc  AGGAACTTCAAGATCCCCAATTCGttccacggggtgaagatgct  TACAAGAAAGCTGGGTtgcaccagcttgcggtaact |

| PA5431-UpF-GWL  PA5431-UpR-Gm  PA5431-DnF-Gm  PA5431-DnR-GWR | TACAAAAAAGCAGGCTgcctatgaacagttgcacgc  TCAGAGCGCTTTTGAAGCTAATTCGtgggtgacgatcacctgttc  AGGAACTTCAAGATCCCCAATTCGtatgtcacgccgtcacacca  TACAAGAAAGCTGGGTctggagtcgtccatccagta |
| --- | --- |
| PA5525-UpF-GWL  PA5525-UpR-Gm  PA5525-DnF-Gm  PA5525-DnR-GWR | TACAAAAAAGCAGGCTagctgaccatggccgagaat  TCAGAGCGCTTTTGAAGCTAATTCGaggatctgcaggaaacgctc  AGGAACTTCAAGATCCCCAATTCGaggcactggaaggactcgaa  TACAAGAAAGCTGGGTtccaggaggttcagc |

^1^Sequences in capital letters are common for all genes amplified and overlap with the Gm or *attB* primer sequences (see Table 1 in main text). Lower-case letters indicate gene-specific sequences used for amplification of genes indicated by their PA annotation number.
